# Malnutrition and Sarcopenia Combined Increases the Risk for Mortality in Older Adults on Hemodialysis

**DOI:** 10.3389/fnut.2021.721941

**Published:** 2021-09-17

**Authors:** Catarina Macedo, Teresa F. Amaral, Juliana Rodrigues, Fernanda Santin, Carla Maria Avesani

**Affiliations:** ^1^Faculty of Nutrition and Food Science, University of Porto, Porto, Portugal; ^2^Graduation Program in Food, Nutrition and Health, Nutrition Institute, Rio de Janeiro State University, Rio de Janeiro, Brazil; ^3^Department of Clinical Science, Intervention and Technology, Karolinska Institutet, Stockholm, Sweden

**Keywords:** chronic kidney disease, hemodialysis, malnutrition, older adults, mortality

## Abstract

**Aim:** Sarcopenia and malnutrition are highly prevalent in older adults undergoing hemodialysis (HD) and are associated with negative outcomes. This study aimed to evaluate the role of sarcopenia and malnutrition combined on the nutritional markers, quality of life, and survival in a cohort of older adults on chronic HD.

**Methods:** This was an observational, longitudinal, and multicenter study including 170 patients on HD aged >60 years. Nutritional status was assessed by 7-point-subjective global assessment (7p-SGA), body composition (anthropometry and bioelectrical impedance), and appendicular skeletal muscle mass (Baumgartner's prediction equation). Quality of life was assessed by KDQoL-SF. The cutoffs for low muscle mass and low muscle strength established by the 2019 European Working group on sarcopenia for Older People (EWGSOP) were used for the diagnosis of sarcopenia. Individuals with a 7p-SGA score ≤5 were considered malnourished, individuals with low strength or low muscle mass were pre-sarcopenic, and those with low muscle mass and low muscle strength combined as sarcopenic. The sample was divided into four groups: sarcopenia and malnutrition; sarcopenia and no-malnutrition; no-sarcopenia with malnutrition; and no-sarcopenia and no-malnutrition. Follow-up for survival lasted 23.5 (12.2; 34.4) months.

**Results:** Pre-sarcopenia, sarcopenia, and malnutrition were present in 35.3, 14.1, and 58.8% of the patients, respectively. The frequency of malnutrition in the group of patients with sarcopenia was not significantly higher than in the patients without sarcopenia (66.7 vs. 51.2%; *p* = 0.12). When comparing groups according to the occurrence of sarcopenia and malnutrition, the sarcopenia and malnutrition group were older and presented significantly lower BMI, calf circumference, body fat, phase angle, body cell mass, and mid-arm muscle circumference. In the survival analysis, the group with sarcopenia and malnutrition showed a higher hazard ratio 2.99 (95% CI: 1.23: 7.25) for mortality when compared to a group with no-sarcopenia and no-malnutrition.

**Conclusion:** Older adults on HD with sarcopenia and malnutrition combined showed worse nutritional parameters, quality of life, and higher mortality risk. In addition, malnutrition can be present even in patients without sarcopenia. These findings highlight the importance of complete nutritional assessment in patients on dialysis.

## Introduction

Chronic kidney disease (CKD) has been recognized as one of the main and most prevalent public health problems worldwide. In fact, in 2017 it was estimated that 1.2 million of people died from CKD with an increase of 41% in the global mortality rate between 1990 and 2017 (1). The global prevalence of CKD is of 9.1% (1), which is similar to the CKD prevalence reported of 8.9% in Brazil in 2015 (2). The main factors justifying this increase in CKD are related to the population aging and to the increase in the prevalence of hypertension, diabetes mellitus, and obesity, which are knows as the main risk factors for the development of CKD (1).

The stages of CKD evolve as the glomerular filtration rate decreases and the clinical condition worsens, requiring renal replacement therapy in the later stages of the disease, with hemodialysis (HD) being one of the therapeutic options. If, on the one hand, HD enables patients with CKD to live, on the other hand, it can contribute to the development of nutritional disturbances malnutrition, sarcopenia, and frailty. Among these patients on HD, malnutrition and sarcopenia stand out due to their high prevalence in dialyzed patients (3).

According to the sarcopenia consensus from the European Working Group on Sarcopenia for Older People (EWGSOP2), sarcopenia is defined as a progressive and generalized skeletal muscle disorder characterized by the occurrence of low muscle strength and low quality or quantity of muscle mass (4). The coexistence of both conditions constitutes the confirmatory criteria for the diagnosis of sarcopenia. Particularly in CKD, sarcopenia has multicausal etiology, with factors that overlap with traditional factors of sarcopenia in the elderly (5). According to a group of experts from the International Society of Renal Nutrition and Metabolism (ISRNM), malnutrition is characterized by multiple changes caused by a set of factors that lead to an increase in protein catabolism, thus leading to a negative protein balance (6). Sarcopenia and malnutrition are highly prevalent in patients on HD, with the former varying from 51 to 68% and the latter varying from 3.9 to 63.3%, depending on the detection method, disease stage, type of treatment, age, and cutoff points used (7). Both conditions are associated with adverse outcomes, including not only decreased quality of life and functionality, but also increased susceptibility to infection, high hospitalization rates, healthcare costs, morbidity, and mortality (8, 9).

Moreover, data from the United States shows that 40% of end-stage patients were over 65 years in 2013, and projections for 2030 indicate that this proportion will increase to 55–61% (10). Given the fast increase in the prevalence of older adults on dialysis in the recent decades, and the effect of senescence on decreasing skeletal muscle mass, the assessment of the outcomes of malnutrition and sarcopenia in patients undergoing chronic HD is of major relevance. Importantly, research has shown that early intervention in these patients increases the quality of life and reduces mortality (11). However, the diagnosis and monitoring of malnutrition and sarcopenia in dialyzed patients is not yet carried routinely out in many dialysis clinics, hindering the early intervention for these two conditions. Although it is well-known that malnutrition and sarcopenia are related to higher mortality risk, we now investigate the effect of these two conditions combined on markers of nutritional status, clinical condition, quality of life, and on mortality.

## Methods

### Study Protocol

This is an observational, longitudinal, and multicenter study including 170 patients under HD treated in six dialysis units in Brazil. A detailed description of the methodology can be found elsewhere (8). All participants were included from March 2010 to February 2014 and were followed for mortality events up to 36 months. Patients who changed dialysis modality or were transferred to other dialysis units or had kidney transplantation were censored.

### Patients

Patients were eligible for inclusion if aged over 60 years, undergoing HD for at least 3 months, three times per week, with each session lasting 3.5–4 h. The exclusion criteria comprised patients using a wheelchair, with amputated limbs and with HIV, cancer, and Alzheimer's and Parkinson's diseases. The study was approved by the Ethics and Research Committee of Rio de Janeiro State University, Brazil registered with protocol number 039.3.2011, and a written informed consent was obtained from all patients before their admission in the study.

### Methods

At baseline, all participants had the nutritional status assessed by the 7-point-subjective global assessment (7p-SGA) translated to Portuguese (12), by anthropometric measurements [body weight, height, midarm circumference, triceps skinfold (SKF) thickness, and hip and calf circumference], bioelectrical impedance (BIA), and handgrip strength (HGS) after 30–60 min the dialysis session in a midweek dialysis day to minimize the influence of fluids overload on body composition (13). SKFs were measured using an SKF caliper (Lange, Cambridge Scientific Industries, Cambridge, MD, USA), following the Lohman's Protocol (14). Body fat was estimated by BIA (Biodynamics® 450; Biodynamics Corporation, Seattle, WA, USA) with the patient in a supine position after 5 min of rest. The measurements of body weight, height, resistance, and reactance were entered in the software Fluid & Nutrition version 3.0 (RJL body composition analyzer) to obtain body fat and phase angle. The study from Heo et al. (15) that evaluated body composition by BIA in non-CKD individuals was used to classify obesity, by using body fat percentage above 32.3% for men and above 44.1% for women. The arm contrary to the arteriovenous fistula was used for the assessment of arm circumference, triceps SKF, and HGS. Muscle strength was measured by a mechanical handgrip dynamometer (Baseline, Fabrication Enterprises, Inc, Elmsford, NY, USA). The highest value of three measurements was taken, with arms along the body after a voice command asking to use the maximal force in the dynamometer. The midarm muscle circumference (MAMC) was calculated using the Frisancho equation (16). The standard values of MAMC and triceps SKF were calculated by the equation: (measured value/value on P50 from NHANES III) × 100 (9).

The 7p-SGA was applied by experienced renal dietitians. The nutritional status of a patient was classified as well-nourished when the 7p-SGA score was equal to 7 and 6 and, as malnourished when the 7p-SGA score ≤ 5 (12).

The Baumgartner's prediction equation (17) was used to estimate appendicular skeletal muscle mass (ASM):


$$\begin{array}{l} ASM\;\left(kg\right)=\;0.2487\;\left(weight,\;kg\right)+\;0.0483\;\left(height,\;cm\right)\\ -\;0.1584\;\left(hip\;circumference,\;cm\right)+\;0.0732\;[HGS,\;kgf\\ \left(kilogram\;force\right)]\;+\;2.5843\;(male)\;+\;5.8828 \end{array}$$

(17)

A previous study, conducted by our research group and which included patients on HD, showed that this equation had good agreement with the ASM assessed by dual-energy X-ray absorptiometry (DXA) with an intraclass coefficient correlation (ICC) of 0.92 (95% CI: 0.86–0.95) (9). The ASM was divided by the square height (m) for calculation of the ASM index (ASMI).

The laboratorial measurements were performed before the dialysis session and included assessment of serum albumin (bromocresol green method), high-sensitive C-reactive protein (hs-CRP; by nephelometry), and 25 hydroxyvitamin D [(25 (OH) D); by chemiluminescence immunoassay]. Serum urea was assessed before and after dialysis session for calculation of the urea Kt/V according to the formula of Daugirdas (18) from a midweek dialysis session.

Quality of life was assessed using the Short Form 1.3 questionnaire (KDQoL-SF) (19), which was applied during the dialysis session in 154 patients from the total sample (170 patients). The reason for a smaller sample having data on quality of life is that this assessment did not start at the beginning of the data collection.

For sarcopenia diagnosis, the cutoffs for low muscle mass and low muscle strength established by the 2019 EWGSOP (4) were used. Low muscle strength was considered when HGS was <27 kilogram force (kgf) for men and <16 kgf for women and low muscle mass was considered when the ASMI was <7.0 kg/m^2^ for male and <5.5 kg/m^2^ for female (4). Patients were considered with pre-sarcopenia when presenting only one of the muscle abnormalities, that is, low muscle mass or low muscle strength and with sarcopenia when both conditions were present.

The patients were classified into four groups considering the presence of malnutrition, pre-sarcopenia, and sarcopenia:

- Group sarcopenia and malnutrition (*n* = 56): Comprised by patients with positive criteria for sarcopenia/pre-sarcopenia and for malnutrition (7p-SGA score ≤ 5).
- Group sarcopenia and no-malnutrition (*n* = 28): Comprised by patients with positive criteria for sarcopenia/pre-sarcopenia, but without criteria for malnutrition (7p-SGA score = 6 and 7).
- Group no-sarcopenia with malnutrition (*n* = 44): Comprised by patients without criteria for sarcopenia/pre-sarcopenia, but with positive criteria for malnutrition (7p-SGA score ≤ 5).
- Group no-sarcopenia and no-malnutrition (*n* = 42): Comprised by patients without criteria for sarcopenia/pre-sarcopenia and for malnutrition (7p-SGA score = 6 and 7).

### Statistical Analysis

The Shapiro–Wilk test was applied to test normality. Categorical variables are described as absolute number and percentage and continuous variables as mean and SD or as median and interquartile range, as appropriate. The comparisons of the variables among the groups of sarcopenia and malnutrition were performed using the chi-square test for categorical variables, and one-way ANOVA or Kruskal–Wallis tests for continuous variables, as appropriate. The Bonferroni test was used to verify the differences among the groups for the variables presenting normal distribution.

The comparisons between the survival and deceased groups were done by chi-square test, independent *t*-test, or Mann–Whitney test, as appropriate. The survival analyses were performed by the Kaplan–Meier graphic using the log-rank test to compare the survival curves among the sarcopenia and malnutrition groups. The Cox's proportional risk model adjusted for gender, age, and hs-CRP was used to assess the hazard ratio for mortality, using the no-sarcopenia and well-nourished group as reference. The value of *p* < 0.05 will be used for statistical significance. All analyses will be performed using the SPSS software version 27 (IBM Corp. Released 2015, Armonk, NY, USA).

## Results

Table 1 shows the main characteristic of the studied sample comprised of older adults on chronic HD. In general, the mean age was around 70.6 years, the majority of the sample was comprised of males, and the urea Kt/V was indicative of adequate dialysis. The mean BMI, calf circumference, standard triceps SKF, and MAMC indicated adequate nutritional status according to cutoffs stablished for non-CKD individuals, which are well-accepted for use in patients with CKD (20). However, when the nutritional status was assessed by 7p-SGA, 58.8% of the sample had a score ≤ 5, indicating malnutrition. As for body composition assessed by BIA, the mean ± SD values for body fat percentage showed that 26.7% of male and 7% of female were obese when applying the cutoffs suggested by Heo et al. (15). When assessing the presence of sarcopenia, about one-third of the sample had either low muscle mass or low muscle strength, here defined as pre-sarcopenia, whereas 14.1% had both conditions combined, defined as sarcopenia, and 50.6% had no signs of low muscle mass or low muscle strength. The laboratory exams were compatible to that observed for patients on dialysis treatment, and the mean serum albumin was within the acceptable values to patients with CKD (>3.8 mg/dl) (21).

**Table 1:** Main demographic, nutritional, and clinical characteristics of older adults on hemodialysis.

	**Results (*n* = 170)**
Age (years)	70.6 ± 7.2
Male (*n*; %)	111 (65.3)
Dialysis length (years)	2.9 (1.3; 5.6)
Urea Kt/V	1.5 (1.3; 1.6)
Diabetes (*n*; %)	44 (37.7)
BMI (kg/m^2^)	25.4 ± 4.5
Pre-sarcopenia (*n*; %)	60 (35.3)
Sarcopenia (*n*; %)	24 (14.1)
No sarcopenia (*n*; %)	86 (50.6)
Standard triceps skinfold thickness (%)	102.5 (72.7; 142.1)
Standard Midarm muscle circumference (%)	98.1 ± 14.7
Calf circumference (cm)	
Male	34.5 ± 3.9
Female	33.3 ± 3.5
Malnutrition (*n*; %)	100 (58.8)
Body fat (%)	
Male	27.5 ± 7.0
Female	37.7 ± 5.3
Phase angle (°)	
Male	5.5 ± 1.3
Female	5.3 ± 1.2
Body cellular mass (kg)	
Male	22.1 ± 4.8
Female	17.0 ± 3.6
Appendicular skeletal muscle mass index (kg/m^2^)	
Male	7.48 ± 0.77
Female	4.60 ± 0.81
Pre-sarcopenia (*n*; %)	
Low HGS	38 (22.4)
Low ASMI	22 (12.9)
Total	60 (35.3)
Albumin (g/dl)	3.9 ± 0.4
Hemoglobin (mg/dl)	11.3 ± 1.6
Hematocrit (%)	34.4 ± 5.0
S Creatinine (mg/dl)	8.7 ± 2.8
S Urea (mg/dl)	138 ± 39.5
PTH (mg/dl)	223 (101; 402)
25(OH)D (ng/ml)	19.2 (12.7; 27.1)
hs-CRP (mg/dl)	0.42 (0.2; 1.1)

*BMI, body mass index; hs-CRP, high-sensitive C-reactive protein; 25(OH)D, 25-hydroxyvitamin D; PTH, parathormone*.

*Data described as absolute values and its percentage for categorical variables; as mean ± SD as or as median and interquartile range for continuous variables according to the variable's distribution. Malnutrition defined as 7p-SGA ≤ 5*.

*Pre-sarcopenia defined as either low muscle mass (appendicular skeletal muscle mass index below 7 kg/m^2^ for male and below 5.5 kg/m^2^ for female) or low muscle strength (handgrip strength below 27 kgf for male and below 16 kgf for female). Sarcopenia was defined by the concomitant condition of low muscle mass and low muscle strength*.

**Figure 1:**
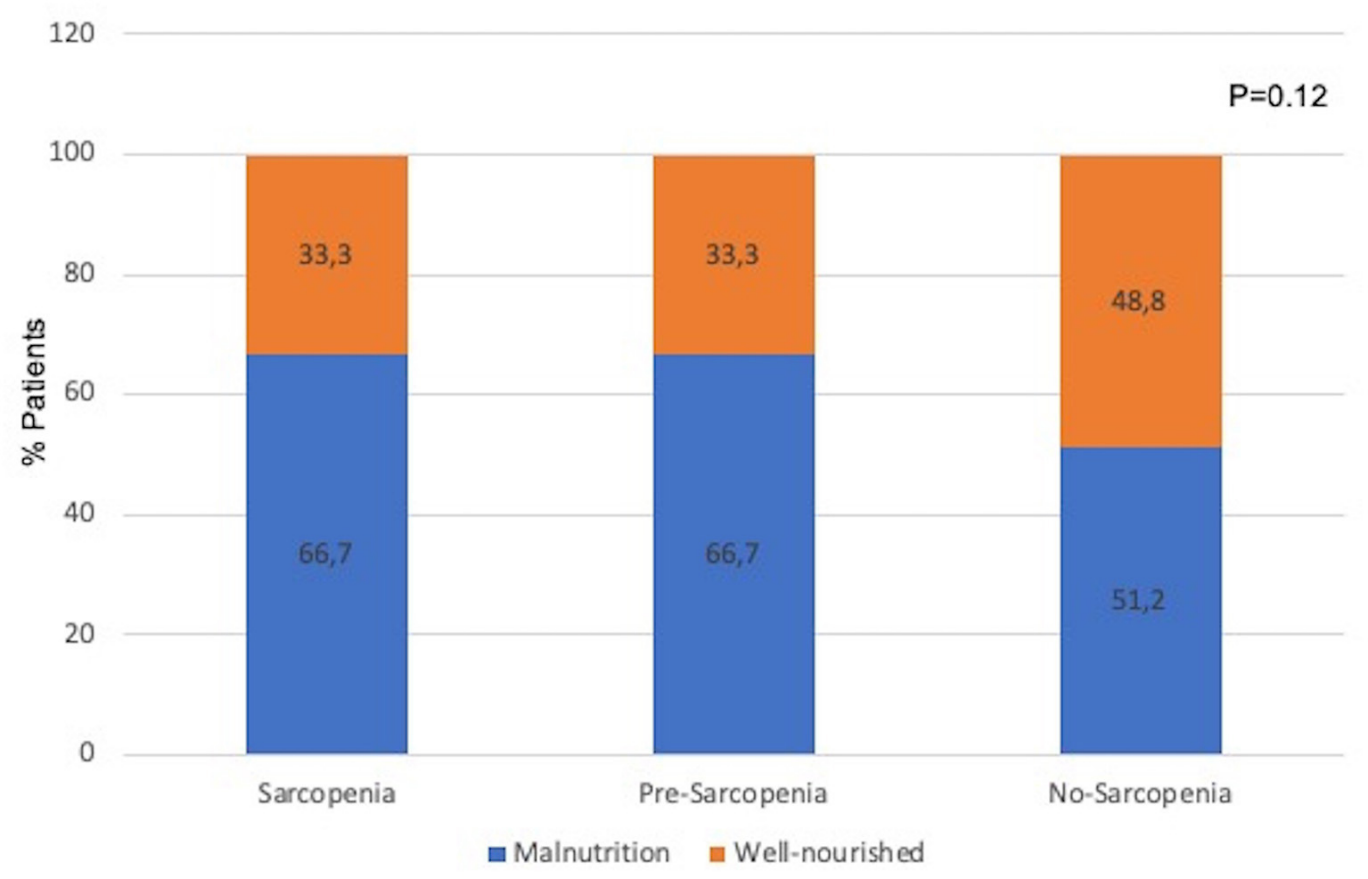
Prevalence of malnutrition (assessed by 7p-SGA) in groups classified as sarcopenia, pre-sarcopenia, and no-sarcopenia. Differences among groups were tested by chi-square test.

Considering that the presence of two nutritional disturbances—malnutrition and sarcopenia were investigated, we evaluated whether the frequency of malnutrition differed among the sarcopenia groups. Figure 1 shows the frequency of patients with malnutrition (assessed as 7p-SGA ≤ 5) in the groups stratified as sarcopenia, pre-sarcopenia, and no-sarcopenia. As can be observed, the prevalence of patients with malnutrition did not differ among the sarcopenia groups, indicating that malnutrition was present even in the group of no-sarcopenia. We then expanded our analysis by exploring the role that these two conditions combined (sarcopenia and malnutrition) have on other nutritional markers, clinical condition, quality of life, and mortality events.

Table 2 shows the comparison of demographic, nutritional, clinical characteristics and quality of life among the groups classified by the presence of sarcopenia and malnutrition. Age and the percentage of males differed significantly among the groups, with the age higher in the group sarcopenia and malnutrition while male gender had higher prevalence in the group sarcopenia and no-malnutrition. Except for serum albumin that did not differ among the groups, the other nutritional markers differed significantly, indicating worse nutritional status in the group sarcopenia and malnutrition as compared to the group no-sarcopenia and no-malnutrition. Regarding clinical condition, the urea Kt/V and 25(OH)D differed among the groups, being the group sarcopenia and malnutrition presenting higher Kt/V and lower 25(OH)D as compared to the group no-sarcopenia and no-malnutrition. Regarding the domains related to the quality of life, most of them did not differ among the groups. Those that showed significant differences were quality of social interaction, role physical, social function, and SF12 mental composite with the group no-sarcopenia and no-malnutrition showing better scores when compared to the remaining groups. After 23.5 (12.2; 34.4) months of follow-up (median and interquartile ranges), there were 62 events of death. The group of deceased patients was older, with higher Kt/V and hs-CRP as compared to the patients who survived (Table 3).

**Table 2:** Comparisons of demographic, nutritional, clinical characteristics, and quality of life of older adults on hemodialysis according to the groups sarcopenia and malnutrition.

	**Sarcopenia and malnutrition (*n* = 56; 33%)**	**Sarcopenia and no-malnutrition (*n* = 28; 16.5%)**	**No-sarcopenia and malnutrition (*n* = 44; 25.8%)**	**No-sarcopenia and no-malnutrition (*n* = 42; 24.7%)**	*p* ^*^
Age (years)	73.2 ± 8.0^a^	72.4 ± 7.9^a, c^	69.3 ± 6.0^b, c^	67.4 ± 5.4^b, d^	<0.001
Male (*n*; %)	34 (60.7)	24 (85.7)	23 (52.3)	30 (71.4)	0.02
Dialysis length (years)	3.01 (1.66; 5.60)	2.96 (1.37; 6.32)	2.08 (0.93; 5;87)	3.15 (1.24; 5.60)	0.67
Urea Kt/V	1.51 (1.40; 1.70)	1.34 (1.22; 1.71)	1.47 (1.30; 1.63)	1.41 (1.28; 1.53)	0.04
Diabetes (*n*; %)	14 (25)	12 (42.8)	24 (54.5)	14 (33.3)	0.06
BMI (kg/m^2^)	23.6 ± 4.7^a^	25.0 ± 3.3^a, c^	25.9 ± 4.6^b, c^	27.7 ± 3.9^b, d^	<0.001
Standard triceps skinfold thickness (%)	89.5 (63.6; 125.7)	110.1 (91.4; 175.7)	96.15 (72.1; 121.2)	134.4 (96.5; 169.1)	<0.001
Standard midarm muscle circumference (%)	93.6 ± 13.4^a^	94.0 ± 13.2^a, c^	100.9 ± 16.7^a, b, c^	103.9 ± 12.7^b^	0.001
Calf circumference (cm)	32.6 ± 3.4^a^	33.7 ± 2.3^a^	33.6 ± 4.6^a^	36.6 ± 2.9^b^	<0.001
Body fat %	28.5 ± 7.8^a^	30.0 ± 8.5^a, b^	32.3 ± 8.2^a, b^	33.9 ± 7.3^b^	0.007
Phase angle (°)	4.9 ± 1.2^a^	5.2 ± 0.9^a, c^	5.4 ± 0.9^a, b, c^	6.2 ± 1.4^b^	<0.001
Body cellular mass (kg)	17.7 ± 4.3^a^	20.0 ± 3.8^b^	19.2 ± 3.8^a, b^	24.2 ± 5.0^c^	<0.001
Appendicular skeletal muscle mass index (kg/m^2^)	6.42 ± 1.1^a^	7.03 ± 0.83^a, b, c^	6.99 ± 0.96^b^	7.56 ± 0.94^c^	<0.001
Albumin (g/dl)	3.83 ± 0.41	3.98 ± 0.40	3.92 ± 0.41	3.91 ± 0.41	0.44
Hemoglobin (mg/dl)	11.3 ± 1.9	11.2 ± 1.7	11.2 ± 1.3	11.4 ± 1.5	0.96
Hematocrit (%)	34.6 ± 6.0	34.3 ± 5.2	34.0 ± 4.1	34.4 ± 4.5	0.94
S Creatinine (mg/dl)	8.5 ± 2.4	8.9 ± 2.9	8.2 ± 2.8	9.4 ± 3.1	0.24
S Urea (mg/dl)	130.6 ± 41.7	150.8 ± 40.9	137.2 ± 37.2	140.4 ± 36.7	0.16
PTH (mg/dl)	165.3 (59.6; 331.6)	218.3 (111.6; 454.1)	262.3 (106.6; 442.8)	256 (171.2; 402.6)	0.23
25(OH)D (ng/ml)	17.5 (11.7; 30.1)	14.7 (10.5; 21.4)	18.0 (13.5; 25.4)	25.7 (19.0; 34.0)	0.001
hs-CRP (mg/dl)	0.37 (0.20; 1.23)	0.26 (0.09; 0.69)	0.52 (0.26; 1.18)	0.52 (0.22; 1.15)	0.19
**Quality-of-life domains**					
Symptom problem list	75.1 ± 16.8	74.5 ± 21.6	67.7 ± 24.0	75.6 ± 18.4	0.24
Effects of kidney disease	68.7 (50.0; 84.4)	71.9 (40.6; 90.6)	59.4 (42.2; 78.1)	75.0 (66.7; 91.7)	0.38
Burden of kidney disease	37.5 (18.8; 56.3)	50.0 (18.8; 75.0)	25.0 (12.5; 50.0)	50.0 (25.0; 68.8)	0.10
Work status	50.0 (0.0; 50.0)	50.0 (0.0; 50.0)	50.0 (0.0; 50.0)	50.0 (0.0; 100.0)	0.83
Cognitive function	80.4 ± 21.0	78.3 ± 26.4	75.9 ± 25.8	83.5 ± 19.4	0.50
Quality of social interaction	81.5 ± 17.9^a^	77.1 ± 20.4^a^	70.7 ± 24.0^a, b^	82.8 ± 19.2^a, c^	0.03
Sexual function	95.8 ± 10.2	85.9 ± 14.5	77.3 ± 26.8	84.2 ± 19.7	0.50
Sleep	65.0 (47.5; 83.8)	57.5 (35.0; 75.0)	57.5 (46.3; 70.0)	72.5 (52.5; 81.3)	0.08
Social support	100.0 (66.7; 100.0)	83.3 (66.7; 100.0)	100.0 (66.7; 100.0)	100.0 (66.7; 100.0)	0.78
Dialysis staff encouragement	75.0 (56.3; 93.8)	87.5 (75.0; 100.0)	100.0 (75.0; 100.0)	87.5 (75.0; 100.0)	0.07
Overall health	60.0 (50.0; 100.0)	50.0 (50.0; 100.0)	60.0 (50.0; 85.0)	60.0 (50.0; 80.0)	0.61
Patient satisfaction	68.4 ± 19.3	75.4 ± 21.2	72.4 ± 21.6	71.9 ± 20.2	0.56
Physical functioning	45.0 (22.5; 80.0)	50.0 (25.0; 70.0)	40.0 (25.0; 70.0)	55.0 (40.0.75.0)	0.21
Role physical	50.0 (0.0; 75.0)	50.0 (0.0; 100.0)	0.0 (0.0; 62.5)	50.0 (25.0; 100.0)	0.009
Pain	62.5 (45.0; 90.0)	70.0 (45.0; 90.0)	55.0 (22.5; 95.0)	67.5 (45.0; 100.0)	0.43
General health	60.0 (30.0; 70.0)	50.0 (35.0; 75.0)	50.0 (32.5; 62.5)	65.0 (40.0; 82.5)	0.15
Emotional well-being	76.0 (52.0; 92.0)	84.0 (60.0; 96.0)	68.0 (42.0; 90.0)	80.0 (64.0; 92.0)	0.18
Role emotional	33.3 (0.0; 100.0)	66.7 (0.0; 100.0)	33.3 (0.0; 66.7)	66.7 (33.3; 100.0)	0.09
Social function	62.5 (37.5; 100.0)	87.5 (62.5; 100.0)	62.5 (25.0; 87.5)	75.0 (62.5; 100.0)	0.04
Energy fatigue	55.0 (32.5; 75.0)	60.0 (40.0; 80.0)	45.0 (32.5; 75.0)	65.0 (42.5; 80.0)	0.27
SF12 Physical composite	37.9 (31.1; 45.8)	36.4 (32.3; 45.3)	36.5 (26.2; 46.5)	40.6 (35.1; 47.2)	0.22
SF12 Mental Composite	44.9 ± 11.1^a^	50.3 ± 13.5^a, b^	44.3 ± 12.3^a^	50.5 ± 10.1^b^	0.02

*NA, non-applicable; hs-CRP, high-sensitive C-reactive protein; 25(OH)D, 25(OH)D, 25-hydroxyvitamin D; PTH, parathormone. Quality of life was evaluated in a subgroup of 154 patients (n = 49, n = 23, n = 41, and n = 41, respectively in the four groups). Data described as absolute values and its percentage for categorical variables; as mean ± SD as or as median and interquartile range for continuous variables according to the variable's distribution*.

* *Chi-square or one-way ANOVA or Kruskal–Wallis test, as appropriate. The Bonferroni post-hoc test for ANOVA p ≤ 0.05: Significant differences among the groups are signed by the different superscript letters*.

**Table 3:** Comparison of older adult patients on hemodialysis according to the group alive and deceased (*n* = 170).

	**Alive (*n* = 108)**	**Deceased (*n* = 62)**	*p* ^*^
Male	71 (65.7)	40 (64.5)	0.87
Age (years)	69.6 ± 6.7	72.5 ± 7.8	0.013
Kt/V	1.42 ± 0.3	1.60 ± 0.5	0.002
hs-CRP	0.34 (0.18; 0.82)	0.58 (0.27; 1.48)	0.004
Dialysis length (years)	2.9 (1.2; 5.4)	2.9 (1.3; 6.0)	0.68

*hs-CRP, high-sensitive C-reactive protein*.

* *The t-test; chi-square test, or Mann–Whitney test, as appropriate*.

**Figure 2:**
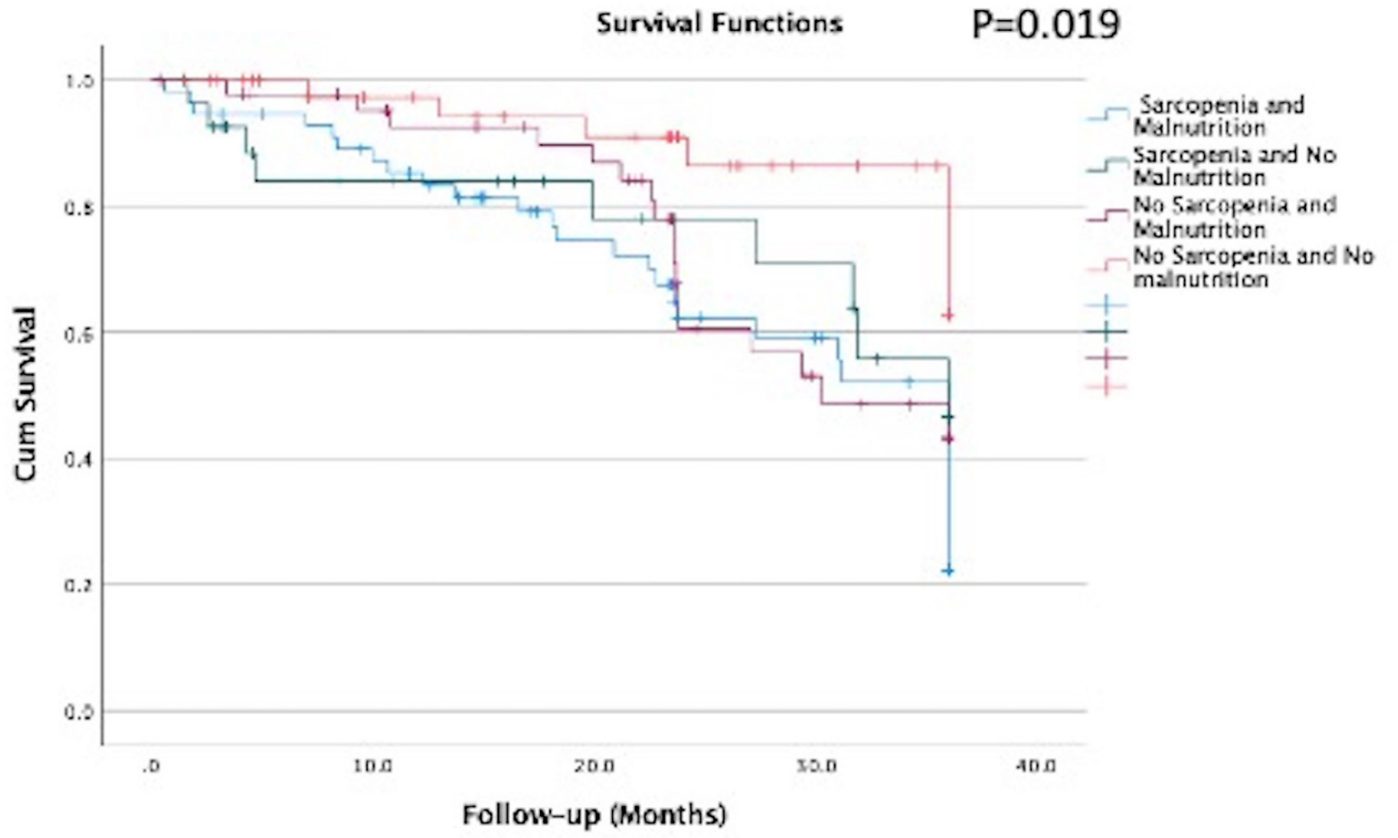
Survival curves according to the groups with sarcopenia and malnutrition of elderly patients on hemodialysis (*n* = 170).

The survival analysis showed that there was a significant difference in the survival curves among the groups, being the group combining both conditions (sarcopenia and malnutrition) the one with lower survival rate (Figure 2, Kaplan–Meier; log-rank test, *p* = 0.019). This finding was confirmed in the Cox regression analysis adjusted for age, gender, and hs-CRP, where the group with sarcopenia and malnutrition had a hazard ratio of 2.99 (95% CI: 1.23: 7.25) as compared to the reference group no-sarcopenia and no-malnutrition (Table 4).

**Table 4:** Risk for mortality events, expressed as hazard ratio^*^ according to the combination of group with sarcopenia and malnutrition in older adults undergoing maintenance hemodialysis (*n* = 170).

		**95% CI**		
	Hazard ratio^*^	**Lower**	**Upper**	***p-*value**
Male	0.44	0.24	0.83	0.01
Age (years)	1.02	0.96	1.06	0.27
hs-CRP (mg/dl)	1.35	2.00	1.58	0.03
No-sarcopenia + no-malnutrition (reference group) (*n* = 42)				
Sarcopenia + no-malnutrition (*n* = 28)	2.65	0.86	7.05	0.09
No-Sarcopenia + malnutrition (*n* = 44)	2.43	0.97	6.05	0.06
Sarcopenia + malnutrition (*n* = 56)	2.99	1.23	7.25	0.03

*hs-CRP, high-sensitive C-reactive protein*.

* *Cox's proportional risk model*.

## Discussion

In this study, we aimed to evaluate the role of sarcopenia and malnutrition on the nutritional markers and survival in a cohort of older adults on chronic HD. Malnutrition (diagnosed by 7p-SGA) was present in 58.8%, which was similar to that found by Cianciaruso et al. (22) where 51% of older adults (>65 years) in HD and peritoneal dialysis had malnutrition assessed by SGA. Moreover, in a meta-analysis aiming to describe the prevalence of malnutrition in patients with CKD (assessed by SGA or malnutrition inflammation score), it was shown that the 25–75th percentile ranges of malnutrition among studies on dialyzed patients was 28–54% (3). Therefore, our findings on the presence of malnutrition are somehow higher than that from previous studies, most likely due to the inclusion of only older adults on HD.

Markers of muscle abnormality, such as low muscle strength or low muscle mass, named as pre-sarcopenia in the current study, were present in 35.3% of the patients, a percentage similar to that found by Isoyama et al. in incident dialyzed patients (39% of the patients with either low muscle strength or low muscle mass) (23). Moreover, we found that sarcopenia (diagnosed by the concomitance of low muscle mass and low muscle strength) was present in 14.1% of the patients, a frequency lower to that from previous studies in dialysis patients (20–40%) (23–25). This discrepancy is most likely due to the diagnostic methods used and the cutoffs applied for the diagnose of low muscle mass and low muscle strength in patients on HD, as previously shown by Lamarca et al. (7). In addition, these divergent results, when comparing with findings from other studies, can also be explained by different characteristics from the studied sample, such as the CKD stage, dialysis modality, presence of comorbidities, and the mean age of the sample (5).

Furthermore, we identified that among the groups stratified by sarcopenia status, 66.7% of the patients from the group sarcopenia had also malnutrition (Figure 2). In another study including older adults (>65 years) with CKD stages 3b to 5 not on dialysis, 52% of the patients with sarcopenia were diagnosed with protein energy wasting (PEW) using the diagnostic criteria from the ISRNM (26). In incident dialysis patients, 64.7% of the patients with sarcopenia had malnutrition diagnosed by SGA (23). The similar frequency of sarcopenia and malnutrition combined found in our study and in the aforementioned ones underlines that these nutritional disturbance can coexist, and a careful assessment for both conditions should be performed in patients on HD.

Surprisingly, when comparing the frequency of malnutrition among the sarcopenia groups (Figure 1), 51.2% of the patients in the group no-sarcopenia had malnutrition, a proportion not different from that observed in the groups sarcopenia and pre-sarcopenia. Similarly, in two previous studies including either patients with CKD stages 3b−5 or before the start of dialysis therapy, 14–20% of the patients with no-sarcopenia had malnutrition (23, 26). In other words, the absence of sarcopenia does not exclude the existence of malnutrition. This finding highlights that although sarcopenia and malnutrition share some common criteria, these are different nutritional abnormalities, and the investigation of both is crucial. In the current study, malnutrition was diagnosed by 7p-SGA, which evaluate several domains of nutritional status (involuntary loss of body weight, food intake, gastrointestinal symptoms, poor appetite, functional status, comorbidities, and physical exam for subcutaneous fat and muscle loss) (27). Therefore, it provides a broad assessment of nutritional status including aspects not included in the criteria for sarcopenia diagnosis. This likely explains the reason why individuals in the group no-sarcopenia had malnutrition when diagnosed by 7p-SGA. Adding to these findings, we also demonstrated, as expected, that when sarcopenia and malnutrition occurred concomitantly (group sarcopenia and malnutrition), all parameters of nutritional status, except for albumin, were worse when compared with the group no-sarcopenia and no-malnutrition. The non-difference in serum albumin among the groups of sarcopenia and malnutrition corroborates the findings from Gama-Axelsson et al. (28) The authors reported that in prevalent dialyzed patients, serum albumin correlated poorly with markers of nutritional status, including SGA score and body composition parameters, but it was significantly correlated with hs-CRP (28). Altogether, this is aligned with the statement from the updated guidelines in nutrition and CKD from the NKF-KDOQI that albumin is a predictor of hospitalization and mortality, and not a marker of nutritional status (29).

It was interesting to note that within the sarcopenia and malnutrition group, the mean values of body fat markers, such as BMI, percentage of body fat, and standard triceps SKFs were within the normal range for non-CKD individuals (15, 30, 31). Similarly, Lee et al. (32) also observed in a group of older adults on HD that patients with low gait speed and low HGS combined had BMI within the normal range. Additionally, in the study from Ren et al. (24), no significant differences were found between no-sarcopenia and sarcopenia in relation to anthropometric indexes, namely TSF, BMI, MAC, and MAMC. The remaining markers of nutritional status differed among the groups stratified as malnutrition and sarcopenia status, being this difference more marked between the group sarcopenia and malnutrition and group no-sarcopenia and no-malnutrition. Among those, the phase angle, which is not much explored in patients on HD, could discriminate adequately the nutritional status in the four studied groups. In our study, the phase angle differed mainly between the sarcopenic and malnourished group and the group no-sarcopenia and no-malnutrition. This finding is in agreement with studies in non-elderly adults on HD, where the phase angle also differed significantly between malnourished and non-malnourished groups (24, 33, 34). Therefore, one marker of nutritional status should not be used alone to evaluate nutritional status, but rather a combination of markers can be used, as in fact stated in the guideline for nutrition and CKD from the NKF-KDOQI (29).

Regarding quality of life, the domains most affected were social interaction, role physical, social function, and SF12 mental composite, which had worse scores in the group sarcopenia and malnutrition. We are not aware of studies in patients with CKD evaluating the role of sarcopenia and malnutrition combined on quality-of-life domains, but in a previous study from our group, we showed that patients on HD with low muscle strength had worse quality-of-life domains than that of the group with low muscle mass (9). Moreover, in another study including patients on dialysis, malnutrition was associated with worse quality of life (35–37) and with the presence of depression and sleep disorders (35).

Finally, when evaluating survival, we found that the mortality risk of the groups with sarcopenia and malnutrition was close to three times higher than the group without any of these abnormalities. As far as we are concerned, there are no previous studies assessing the role of malnutrition and sarcopenia combined in older adults on HD, but studies in older adults hospitalized without CKD, showed that older adults with combined sarcopenia and malnutrition had a risk for mortality of close to five times higher when compared to the group with none of these nutritional disturbances (38). In patients on dialysis, previous studies have consistently shown that malnutrition (36, 39), sarcopenia (25), and low muscle strength (23, 32, 40) were associated with increased mortality.

Some limitations and strengths of this study can be listed. As a limitation, the observational study design can impair the identification of a causality–effect association. Second, the relatively small sample size can underpower the comparison among the sarcopenia and malnutrition groups, though statistical differences were already listed with this sample size. Third, the lack of robust methods to estimate muscle mass can hinder muscle abnormalities related to muscle mass. As positive aspects, we consider the originality of evaluating the concomitance of malnutrition and sarcopenia in elderly patients on HD, and the relationship of these conditions with quality of life and survival. In addition, although the methods used to evaluate muscle mass could be influenced by the variation in the hydration status, all measurements were performed after the dialysis session to minimize the influence of fluid retention. In addition, since these are the methods used in the routine care of dialysis clinics and also recommended by the updated guidelines in nutrition and CKD from NKF/KDOQI (29), our findings can be used to support a nutritional assessment with methods that are suitable for the routine use.

In conclusion, patients on HD aged 60 years and older who have sarcopenia and malnutrition showed worse nutritional parameters, quality-of-life domains, and higher mortality risk. In addition, we reported that malnutrition can occur in patients without sarcopenia, and that the body fat markers within the normality range can occur concomitantly with malnutrition and sarcopenia. Altogether, these findings highlight the importance of complete nutritional assessment in older patients on dialysis. Further studies to better understand the role of these abnormalities in the health of older adults undergoing maintenance HD are needed.

## Data Availability Statement

The raw data supporting the conclusions of this article will not be made available by the authors.

## Ethics Statement

The studies involving human participants were reviewed and approved by Ethics and Research Committee of Rio de Janeiro State University, Brazil. The patients/participants provided their written informed consent to participate in this study.

## Author Contributions

CM and TA analyzed and interpreted the data, drafted the article, and gave final approval of the submitted version. JR and FS acquired the data, critically revised the important intellectual content, and gave final approval of the submitted version. CA conceived and designed the study, acquired, analyzed, interpreted the data, drafted the article, critically revised the important intellectual content, and gave final approval of the submitted version. All authors contributed to the article and approved the submitted version.

## Funding

This research received two grants from Fundação Carlos Chagas Filho de Amparo à Pesquisa do Estado do Rio de Janeiro (FAPERJ; Grant Numbers: E-26/111.653/2010 and E-26/103.209/2011).

## Conflict of Interest

The authors declare that the research was conducted in the absence of any commercial or financial relationships that could be construed as a potential conflict of interest.

## Publisher's Note

All claims expressed in this article are solely those of the authors and do not necessarily represent those of their affiliated organizations, or those of the publisher, the editors and the reviewers. Any product that may be evaluated in this article, or claim that may be made by its manufacturer, is not guaranteed or endorsed by the publisher.
